# Neuroscience in Education: A Bridge Too Far or One That Has Yet to Be Built: Introduction to the “Brain Goes to School”

**DOI:** 10.3390/brainsci13010040

**Published:** 2022-12-24

**Authors:** Gerry Leisman

**Affiliations:** 1 Movement and Cognition Laboratory, Department of Physical Therapy, University of Haifa, 19 Abba Khoushy Avenue, Eshkol Tower, Rm. 910, Haifa 3498838, Israel; g.leisman@alumni.manchester.ac.uk; Tel.: +972-52-420-5643; 2 Department of Neurology, University of the Medical Sciences of Havana, Havana 11600, Cuba

**Keywords:** developmental cognitive neuroscience, educational neuroscience, neuroeducation

## Abstract

There have been numerous detractors and supporters relating to attempts to merge the neurosciences and the knowledge base of related contributing disciplines with the field of education. Some have argued that this is a “bridge too far”. The predominant view is that the relationship between neuroscience and the classroom has been neither significantly examined, nor applied. What is needed is a specially trained class of professionals whose role it would be to guide the introduction of cognitive neuroscience into educational practice in a sensible and ethical manner. Neuroeducators would play a pivotal role in assessing the quality of evidence purporting to be relevant to education, assessing who is best placed to employ newly developed knowledge, as well as with what safeguards, in addition to investigating how to deal with unexpected consequences of implemented research findings. This special issue of the “The Brain Goes to School” aims to provide support for the development of training programs that truly integrate curriculum design and classroom instruction with the developmental cognitive neurosciences.

Educational neuroscience is a promising area of study, which can collectively integrate the knowledge base found in developmental cognitive neuroscience, [1,2] cognitive science and cognitive neuroscience, [3], psychology, [4] educational theory, [5] human factors, [6] production management, [7,8] educational technology, [9] curriculum design, [10] and even architecture [11,12]. The penultimate aim is to develop basic and applied research that can support the development of practical applications for more effective instruction and learning. 

There have been numerous detractors and supporters of attempts to merge the neurosciences with the knowledge base of the contributing disciplines. In a 2004 paper, Davis [13] noted that “medical models” of cognition, “...have only a very limited role in the broader field of education and learning mainly because learning-related intentional states are not internal to individuals in a way which can be examined by brain activity.” On the other hand, Pettito and Dunbar [14,15] have indicated that educational neuroscience “Provides the most relevant level of analysis for resolving today’s core problems in education.

Several educational researchers have held that merging the knowledge base of developmental cognitive neuroscience with education is a “bridge too far” [16,17], and others have argued against such a view [18,19,20,21]. Nevertheless, a bridging discipline, such as educational or cognitive psychology [22,23], can provide a neuroscientific basis for educational practice. The predominant view, however, seems to be that the relationship between neuroscience and the classroom has neither been significantly examined nor applied [24,25,26].

In some way or another, the brain sciences ought to be able offer much for the learner, given the vast literature on the psychology of learning [27], neuroscience of learning [28], human factors and information processing [29], and developmental cognitive neuroscience [30]. Educational neuroscience has been “birthed” from the necessity to translate practical applications of the knowledge base of these and other related disciplines within classroom applications.

One fundamental difference between the findings of the psychology and neuroscience of learning and classroom instruction is that we, as organisms, can extract, by interaction, data and information from our external environment [31]. Alternatively, school-based instruction explains and describes information and provides data and facts on material that the learner cannot acquire on his or her own. Therefore, the science of learning and the science of education are disparate but complementary. What education as a discipline has largely not considered are genetic, brain state, and endocrine functions that can significantly impact the school-based learning processes both positively and negatively. Educational neuroscience, as a discipline, has the potential to offer much to the education of children and adults, but a fundamentally better understanding of its potential for theory, practice, and curriculum development is in its infancy, and so the justification for the special issue of “The Brain Goes to School” is expounded upon here. The same can also be said for the field of developmental cognitive neuroscience [22,32].

Numerous researchers have taken opposite or middle positions on whether developmental cognitive neurosciences can be linked with education. Bruer [16] had created a significant response when noting that the integration of brain sciences and education is not possible and that some translational field is necessary to support the development of a common language. Goswami [33] indicated that developmental cognitive neuroscience has already effected change, with examples that she provides, which have included “neural markers” that could be employed to assess achievement of developmental educational indicators.

Potential application areas include the investigation of brain processes involved in mathematics instruction and understanding [34], reading [35,36], sustained attention [36], attention deficit hyperactivity disorders [37,38], memory and retention [38,39,40], and many other areas. The knowledge base that already exists but with little serious study in translational research in education includes: language, mathematics, attention and executive control, social and emotional cognition, information processing, movement sciences, production management and industrial engineering, human factors related to psychology and engineering, architecture and classroom design, video gaming, neuroplasticity, developmental and cognitive neurosciences, and a plethora of other factors. It is not simply a matter of putting a neuroscientist in a classroom in a teacher training college, but rather, it is pertinent to how to educate a neuroscientist about classroom needs and then design studies addressing those specific needs. Interdisciplinary degree programs need to be precisely that, with instructors in educational training programs in a classroom all at the same time being neuroscientists. With that kind of thinking, we may be able to design more effective study protocols that truly address concerns of both child and adult learners and perhaps even influence educational policy from evidence and science-based perspectives.

Willingham [41] highlights three challenges that must be overcome to marry the two disciplines effectively. The goals problem entails the following. Willingham suggests that education is an “artificial science’ construct with the “artifacts” of pedagogic strategies and materials. Neuroscience, on the other hand, being a “natural science”, is concerned with the description of neural systems. The consequent result is that education’s goals are difficult to achieve with neuroscience methods, with examples possibly being art or music appreciation. The vertical problem, according to Willingham, suggests that mapping of brain structure and function onto cognition is the highest level of analysis for neuroscientists. It is claimed that there is less room for context or how rote learning may be most effective in the laboratory, but not necessarily so in the classroom, as motivation has not been considered. The horizontal problem, presented by Willingham, indicates that education theories are largely behavioral, but that neuroscientific findings are more multifaceted (e.g., electrochemical, spatial, temporal, etc.).

Willingham suggests that what is essential for a successful union of neuroscience and education is that both fields should have realistic expectations of one another. For example, educators should not expect that neuroscience will provide prescriptive answers for educational practice or answers for educational goals that are incompatible with neuroscientific methods (e.g., aesthetic training) or levels of analysis beyond the individual level. Finally, Willingham suggests that neuroscience will only be useful to educators when targeted at a specific problem at a fine-grained level of analysis, such as how people read, but that these data will only be useful in the context of well-developed behavioral theories that directly relate to curriculum design and instructional methods.

Another review of the educational neuroscience debate focused on scientific and practical challenges, with an emphasis on merging developmental cognitive neuroscience with educational practice [42]. These challenges revolve around: (a) methodological issues in which the neurosciences create contrived environments and thus cannot yield information appropriate for the classroom. The argument continues with the notion that, if neuroscience influences educational practice, contextual variables may be concomitantly deemphasized. The result would then be that the solutions to educational problems could become primarily biological, rather than instructional. (b) Knowing the brain region that supports an elementary cognitive function tells us nothing about how to design instruction for that function. However, Varma et al. [42] suggest that neuroscience provides the opportunity for a novel analysis of cognition, breaking down behavior into elements invisible at the behavioral level. We could, for example, investigate not the brain areas involved in a particular task, but rather the nature of the networks, and then examine how efficient or optimized the problem-solving strategy employed was while also allowing for effective comparisons between different cognitive systems. (c) Educational neuroscience has been accused of developing reductionist theories with the results being of no practical use to educators. Varma et al. had indicated that reductionism is a mode facilitating the unification of ideas. Varma and colleagues have indicated that the co-opting of neuroscience does not necessarily require the eradication of education terminology, but simply provides the opportunity for interdisciplinary communication and understanding. (d) Education and neuroscience are essentially incompatible and illogical, as one cannot describe classroom behavior by methods that measure the physical mechanisms of a learner’s brain. However, neuroscience has the capacity to aid in the resolution of theoretical conflicts within education resulting from differing theoretical constructs in education.

What is needed are individuals trained as professional neuroeducators, a specially trained class of professionals whose role would be to guide the introduction of cognitive neuroscience into educational practice in a sensible and ethical manner. Neuroeducators would play a pivotal role in assessing the quality of evidence purported to be relevant to education, assessing who is best placed to employ newly developed knowledge, and with what safeguards, as well as how to deal with unexpected consequences of implementing research findings [43]. This is precisely what we propose here in these pages and in the development of training programs that truly integrate curriculum design and classroom instruction into the developmental cognitive neurosciences.
